# Return Migration Selection and Its Impact on the Migrant Mortality Advantage: New Evidence Using French Pension Data

**DOI:** 10.1215/00703370-10938784

**Published:** 2023-10-01

**Authors:** Michel Guillot, Myriam Khlat, Romeo Gansey, Matthieu Solignac, Irma Elo

**Affiliations:** Population Studies Center, University of Pennsylvania, Philadelphia, PA, USA; French Institute for Demographic Studies, Aubervilliers, France;; French Institute for Demographic Studies, Aubervilliers, France;; The World Bank, Washington, DC, USA; University of Bordeaux, Bordeaux, France; French Institute for Demographic Studies, Aubervilliers, France;; Population Studies Center, University of Pennsylvania, Philadelphia, PA, USA;

**Keywords:** Immigration, Mortality, Migrant mortality advantage, Return migration, France

## Abstract

The migrant mortality advantage (MMA) has been observed in many immigrant-receiving countries, but its underlying factors remain poorly understood. This article examines the role of return migration selection effects in explaining the MMA among males aged 65+ using a rich, unique dataset from France. This dataset contains information on native-born and foreign-born pensioners who are tracked worldwide until they die, providing a rare opportunity to assess return migration selection effects and their impact on the MMA. Results provide evidence of substantial and systematic negative return migration selection among foreign-born males in France. Old-age returns, in particular, appear particularly affected by such selection; however, they are not frequent enough to explain the MMA at ages 65+. By contrast, returns at younger ages are much more frequent, and the MMA at ages 65+ essentially disappears once these earlier returns are considered. This study extends the literature on negative selection at return and its impact on the MMA by providing evidence that such negative selection may operate not only at older ages but throughout the life course, with impacts on the MMA that are larger than previously suggested.

## Introduction

In the last decades, immigrant populations have increased in both absolute and relative size in many receiving countries. Between 1990 and 2020, the proportion of immigrants in the total population increased from 23.3% to 30.1% in Australia, 15.7% to 21.3% in Canada, 10.4% to 13.1% in France, 7.5% to 18.8% in Germany, 9.2% to 19.8% in Sweden, 6.4% to 13.8% in the United Kingdom, and 9.2% to 15.3% in the United States (United Nations 2020). Given these trends, health and mortality patterns among immigrants are of increasing importance for receiving countries because they affect the demand for health care, health insurance schemes, pension systems, and mortality estimates (Hendi and Ho 2021; Rechel et al. 2013; Zallman et al. 2013). Understanding health and mortality patterns among immigrants is also relevant for social policies aimed at reducing health disparities.

In the literature on immigrant mortality in high-income countries, the most systematic finding is that immigrants tend to exhibit lower mortality than the native population of their receiving country, a phenomenon termed the *migrant mortality advantage* (MMA). This pattern has been reported in receiving countries as diverse as Australia (Kouris-Blazos 2002), Belgium (Deboosere and Gadeyne 2005), Canada (Bourbeau 2002), France (Boulogne et al. 2012; Khlat and Courbage 1996), Germany (Razum et al. 1998), the Netherlands (Bos et al. 2004), Switzerland (Tarnutzer et al. 2012), the United Kingdom (Wallace and Kulu 2014), and the United States (Ruiz et al. 2013; Zheng and Yu 2022). The MMA is often referred to as a *mortality paradox* because it occurs despite the lower average socioeco nomic status (SES) among many immigrant groups relative to the native population of their receiving country.

Several hypotheses have been proposed to explain the MMA, although the relative contribution of each hypothesis in various contexts remains highly debated in the literature (Agyemang 2019; Markides and Eschbach 2011). First, the hypothesis regarding in-migration selection effects (sometimes referred to as the *healthy migrant effect*) posits that individuals who leave their country of origin may be positively selected on health. Second, cultural factors are hypothesized to produce more favorable health behaviors among immigrants in receiving countries than among natives and perhaps also benefit immigrants through dense immigrant social networks. Third, the MMA is hypothesized to result from data artifacts arising from the difficulty of correctly tracking immigrant populations in receiving countries and their corresponding deaths.

Finally, a hypothesis regarding return migration selection effects, sometimes referred to as the *salmon bias* hypothesis and most often discussed in reference to older ages, postulates that immigrants who are in poor health (or with other characteristics associated with higher mortality) may be more likely to return to their country of origin than their healthier counterparts (Abraido-Lanza et al. 1999; Arenas et al. 2015; Di Napoli et al. 2021; Hummer et al. 2007; Khlat and Darmon 2003; Markides and Eschbach 2011; Pablos-Méndez 1994; Palloni and Arias 2004; Palloni and Morenoff 2001; Shai and Rosenwaike 1987; Turra and Elo 2008). Because of this “unhealthy remigration,” immigrants who remain in the receiving country may be selectively healthier, leading to reported mortality rates that are artificially low and poorly reflect the overall health conditions of immigrants.

In this study, we examine return migration selection effects and their impact on the MMA using a rich, unique dataset from France. This dataset contains information on pensioners from France’s largest pension fund who receive lifelong annuities based on contributions accumulated while working in France. These annuities are not contingent on residing in a particular country; pensioners in this dataset are thus tracked worldwide until they die. These data include both native-born and foreign-born pensioners, providing a rare opportunity to assess return migration selection effects and their impact on the MMA. For substantive and methodological reasons (explained later in the article), our analyses focus on males aged 65+.

## Background

### Return Migration Selection Bias, Censoring Bias, and the Salmon Bias

Return migration selection is often raised in the literature as a factor contributing to the migrant mortality advantage (Namer and Razum 2018). There are several theoretical reasons to believe that return migrants may be subject to negative health selection, defined here as a negative correlation, at a given age, between health status and the likelihood of returning to the country of origin. First, studies have shown that immigrants often express a desire to die in their country of origin (Razum et al. 2005; Tezcan 2019). Immigrants may respond to this desire in a way that does not involve health selection—for example, by setting age targets for their return, irrespective of health status. Deterioration of health, however, may trigger or accelerate the decision to return, thereby producing negative health selection. Other reasons for unhealthy remigration include seeking family support (Pablos-Méndez 1994) or more affordable health care in the country of origin (Arenas et al. 2015). Also, for working-age immigrants, a health deterioration can negatively affect employment and income prospects in the receiving country, lowering the financial incentives to remain (Durand 2006).

Negative health selection at return may also operate indirectly if immigrants with low SES, which is associated with higher mortality, are more likely to return than immigrants with high SES, which is associated with lower mortality. Negative health selection could thus occur even when the decision to return is not directly related to health (Razum et al. 1998). Such negative remigration selection is predicted by neoclassical economics theory, which views return migrants as failures or mistaken migrants—that is, migrants whose wages in the receiving country were lower than expected or who experienced higher than anticipated psychological costs of living abroad (Constant and Massey 2002; Duleep 1994; Durand 2006; Wassink and Hagan 2018).

Negative health selection at return, whether operating directly via health or indirectly via SES, mechanically makes the reported mortality of immigrants in a receiving country lower than would be observed in the absence of return migration. This out-migration selection effect is equivalent to the classic problem of informative censoring in event-history analysis, a problem known to produce biased estimates of hazard rates. Because of their connection with health deterioration or, at the extreme, the desire to die in the country of origin, unhealthy remigrations are usually considered particularly salient at older ages, when poor health prevalence increases (Palloni and Arias 2004; Turra and Elo 2008).

Despite theoretical support for negative return migration selection, poor health could also be theorized to *lower* the likelihood of returning, depending on individual and contextual circumstances. For example, the receiving country might have more accessible and higher quality health care than the country of origin, or family support might be more available in the receiving country (Massey et al. 2015; Van Hook and Zhang 2011). As for indirect health selection via SES, new economics theory predicts that return migrants are positively selected on earnings; such theory views return migrants as target earners who return home once they have met their earnings targets (Constant and Massey 2002; Wassink and Hagan 2018). This theory sees return migrants as successes and as subject to positive selection forces. Therefore, the existence of negative selection at return should not be presumed and ultimately depends on the net effect of various forces operating in opposite directions.

Return migration can also contribute to biased mortality rates among immigrants via *censoring bias*, which occurs when data sources in the receiving country fail to correctly remove out-migrants from the risk pool. Because immigrants who leave the receiving country are no longer covered by the receiving country’s vital registration system, a failure to censor them makes them statistically immortal. In other words, censoring bias artificially inflates the population at risk, generating mortality estimates that are too low. Censoring bias is distinct from return migration selection effects: a failure to censor out-migrants biases migrant mortality rates even in the absence of return migration selection (Palloni and Arias 2004; Wallace and Kulu 2018). Censoring bias is best categorized as a data quality explanation for the MMA that could theoretically be addressed by properly tracking and censoring out-migrants.

The salmon bias hypothesis, as originally formulated by Pablos-Méndez (1994), covers both types of biases. This hypothesis postulates that out-migrations are both selective and not correctly recorded in data sources—two biases that combine to downwardly bias reported mortality rates among immigrants in receiving countries. Although most of the subsequent literature uses this definition of the salmon bias, some studies use a narrower definition that focuses more specifically on selection effects (Turra and Elo 2008). In our study, the availability of information on out-migrations allows us to address censoring bias and thereby to focus specifically on return migration selection and its impact on the MMA.

### Evidence for Return Migration Selection Bias

Studies seeking to identify return migration selection effects have produced inconsistent conclusions. Earlier studies relied on indirect evidence, comparing mortality levels among immigrant groups with different return migration patterns. Focusing on Hispanic individuals in the United States, Abraido-Lanza et al. (1999) found that Cuban immigrants had relatively low levels of reported mortality, evidence contradicting the salmon bias hypothesis. By contrast, Palloni and Arias (2004) found that Mexican immigrants in the United States experienced an unusually small slope of mortality at older ages, which was interpreted as evidence of the salmon bias. Other indirect evidence for return migration selection involved analyses of the health status of current versus return migrants using binational surveys. Palloni and Arias (2004) and Riosmena et al. (2013) compared health status among foreign-born Mexicans in the United States versus Mexicans in Mexico with prior U.S. migration experience. Both studies found that the latter group had worse health, evidence consistent with negative return migration selection.

A few studies have sought to capture health selection more directly by examining whether the health status of immigrants in the receiving country was correlated with subsequent out-migration rates. Studies from the United States (Arenas et al. 2015) and the United Kingdom (Wallace and Kulu 2018) found some evidence that immigrants in poor health had higher probabilities of return. By contrast, a study by Diaz et al. (2016) focusing on Mexican return migrants from the United States produced mixed support for negative health selection.

In this literature, a study by Turra and Elo (2008) using U.S. Social Security data stands out for its ability to observe immigrants’ mortality *after* their return to their country of origin. Their results showed that foreign-born Hispanic individuals who returned abroad had higher mortality than those who stayed in the United States, consistent with negative return migration selection. They concluded, however, that the observed amount of negative selection was not sufficient to explain the Hispanic mortality advantage. (For a study focusing on internal migration in Sweden, with results consistent with salmon bias effects, see Andersson and Drefahl (2017).)

The French pension data we use in our study shares many features with the U.S. Social Security data Turra and Elo (2008) used, including direct information on return migration and subsequent mortality. These shared features provide a unique opportunity to apply an approach similar to that of the Turra and Elo study to the European context. We focus on France, one of the major receiving countries in Europe. Relative to the Turra and Elo study, we have information from a more diverse set of countries of origin of immigrants, and we draw on this diversity to interpret our results. More broadly, we expand the literature on negative selection at return and its impact on the MMA by providing evidence that negative selection operates not only at older ages but throughout the life course, with impacts on the MMA that are larger than previously suggested.

### The French Immigration Context

Although not considered a traditional country of immigration (e.g., the United States, Canada, or Australia), France stands out as the oldest European immigrant-receiving country (since the mid-nineteenth century) and the one that has received the largest cumulative number of immigrants (Noiriel 1988; Weil 2005). Before 1945, migration flows to France involved primarily migrants from European countries (Italy, Spain, Portugal, Belgium, and Poland). After 1945, large waves of colonial migrants arrived (mostly from North Africa). Despite a labor migration decrease after 1973 (Therborn 1987), immigration to France continued, mostly via family reunification and asylum (Wihtol de Wenden 2012). In addition, the diversity of migrants continued to increase, with larger proportions of migrants from sub-Saharan Africa and Asia. The creation of the borderless Schengen area in 1995 and the European Union (EU) expansion in 2004–2007 also increased immigration from other EU countries.

In 2021, the proportion of immigrants in France’s population was 12.8% (10.3% if we remove individuals born abroad with a French nationality at birth, who are not technically immigrants per France’s official definition) (INSEE 2022a). The two most important regions of origin for France’s immigrants were North Africa (Algeria, Morocco, and Tunisia) and southern Europe (Italy, Portugal, and Spain), representing 29.3% and 16.2% of the total immigrant population, respectively (INSEE 2022a). The proportion of immigrants from southern Europe is larger among the elderly population, the focus of this study. In 2021, these immigrants represented 33.7% of all immigrants aged 60 or older (vs. 29.3% for immigrants from North Africa), reflecting their preponderance during earlier waves of immigration (INSEE 2022b). The presence in France of elderly immigrants from mainly two different regions—southern Europe and North Africa—with different mortality environments and different border regimes (i.e., southern Europe is part of the borderless Schengen area, whereas North Africa is not) offers useful analytical contrasts for this study.

## Data and Methods

### Data

This study uses longitudinal data from the Caisse Nationale d’Assurance Vieillesse (CNAV, or National Old-Age Insurance Fund), France’s most important pension fund. The CNAV manages pension payments for all individuals who have ever been employed in the private sector in France, regardless of the work contract length. The CNAV maintains a database that tracks pensioners from when they start receiving their pension until their death. This database has high coverage of the elderly male population in France. Approximately 95% of all male French pensioners receive at least a fraction of their pension from the CNAV (Aubert 2012) because it is rare for individuals whose careers were mainly in other sectors (e.g., public sector, self-employed) not to have ever worked in the private sector at some point—for example, via short-term private contracts. Coverage is just as high among foreign-born male pensioners as their native-born counterparts (Aubert 2012; INSEE 2012). However, coverage is lower for females, especially foreign-born females. Certain groups of foreign-born women have particularly low rates of labor force participation, including women born in North Africa and Turkey, whose labor force participation rates are 61% and 50%, respectively (vs. 88% for native-born women) (Perrin-Haynes 2008). These low rates of labor force participation raise concerns about the lack of representativeness of foreign-born elderly women in the database. Our study therefore focuses on males.

We use data on a stratified random sample taken from the CNAV’s exhaustive pensioners database and provided by the CNAV. This sample includes males who were alive at the baseline date of January 1, 2009, and were receiving at least part of their pension from the CNAV as primary beneficiaries. (We excluded secondary beneficiaries, who derive their entire pension based on their spouse’s work history, because they may have never lived in France.) The sample was not restricted by residence; our pensioners may have resided in France or abroad at baseline. Given our focus on immigrants, the CNAV prepared a dataset that oversampled foreign-born individuals, providing us with an original sample of 194,083 foreign-born and 45,561 native-born male pensioners. The dataset contains information on date of birth and country of birth, as well as full residence and mortality follow-up for six calendar years (i.e., January 1, 2009–December 31, 2014). The residence follow-up includes information on the country of residence, updated quarterly based on the pensioner’s reported address changes. The mortality follow-up includes death dates for pensioners who died during the follow-up period. The follow-up information also includes pension amounts the pensioners received each year. Because the dataset does not include information on nationality at birth, we define immigrants based on their country of birth.

We excluded all individuals younger than 65 at baseline (45,561 individuals) to address concerns about early retirement–related health selectivity. We also excluded 1,076 individuals (mostly German-born) who derived their pension not from having worked in France but from their prior association with the French military, perhaps without ever having resided in France. Our analysis sample consists of 193,007 individuals: 160,412 foreign-born and 32,595 native-born.

Because the data are administrative, our sample contains little missing information, although quarterly information about the place of residence was sometimes missing. There were 2,523 pensioners (1.31% of the analysis sample) with missing residence information in at least one quarter. Although the number of quarters for which data were missing during the observation window varied across pensioners, approximately 97.6% of all missing cases had three or fewer quarters with missing residence information. We imputed missing residence information according to systematic rules using the sequence of residence data, survival status, and place of death for those who died during the observation window. Appendix Table A1 (online appendix) summarizes our procedure for imputing missing residence information.

#### Adjustment of Deaths Occurring Abroad

For deaths occurring in France, death information is automatically integrated into the CNAV database via the civil registration system. For deaths occurring abroad, except for a few EU countries with data-sharing agreements (e.g., Belgium, Luxembourg, and Germany), no such integration exists. The recording of death information requires the provision of an official death certificate from the country where the death occurred. Because this integration is not automatic, some deaths occurring abroad may be missing. To reduce the likelihood that pension payments will continue after a pensioner’s death, pensioners residing abroad are required to submit a “certificate of life” to the CNAV every year. This form, which must be certified by local authorities after the presentation of proper identification, proves that pensioners residing abroad are alive and allows them to continue receiving their pension. The CNAV assumes that pensioners who stop producing this certificate are dead and thus stops sending pension allowances.

Given these concerns about tracking deaths occurring abroad, a sole reliance on official death certificates is insufficient for our study. We thus take advantage of the CNAV’s tracking procedure via certificates of life to adjust deaths occurring abroad. Specifically, we examine sequences of annual pension allowances and identify pensioners residing abroad who stopped receiving their pension without official information that they had died. We assume that individuals who stopped receiving their pension allowance at some point during the six-year follow-up period and did not receive it again by the end of the period were no longer alive. To avoid overcorrecting, and given the occurrence of one-year gaps in pension payments in the database, we take the conservative approach of imputing deaths only for individuals with at least two consecutive years without pension allowance. This approach required having at least two consecutive years of pension data to decide about death imputation; we therefore discarded the last two years of data and restricted the analysis window to January 1, 2009—December 31, 2012 (four years). Our imputation strategy adds 959 deaths to the recorded 12,917 deaths occurring abroad during the observation window. Details about the number of imputed deaths corresponding to each pension sequence are summarized in Table A2 (online appendix).

#### Simplifying Residence Information

Most commonly, pensioners did not change residence during the observation period or experienced only one international move. Few pensioners (389, or 0.2% of the sample) experienced two or more changes of residence. To include these individuals in the analysis, we simplified their migration histories by allowing no more than one change of residence during the follow-up period, using the last observed change of residence.

**Fig. 1:**
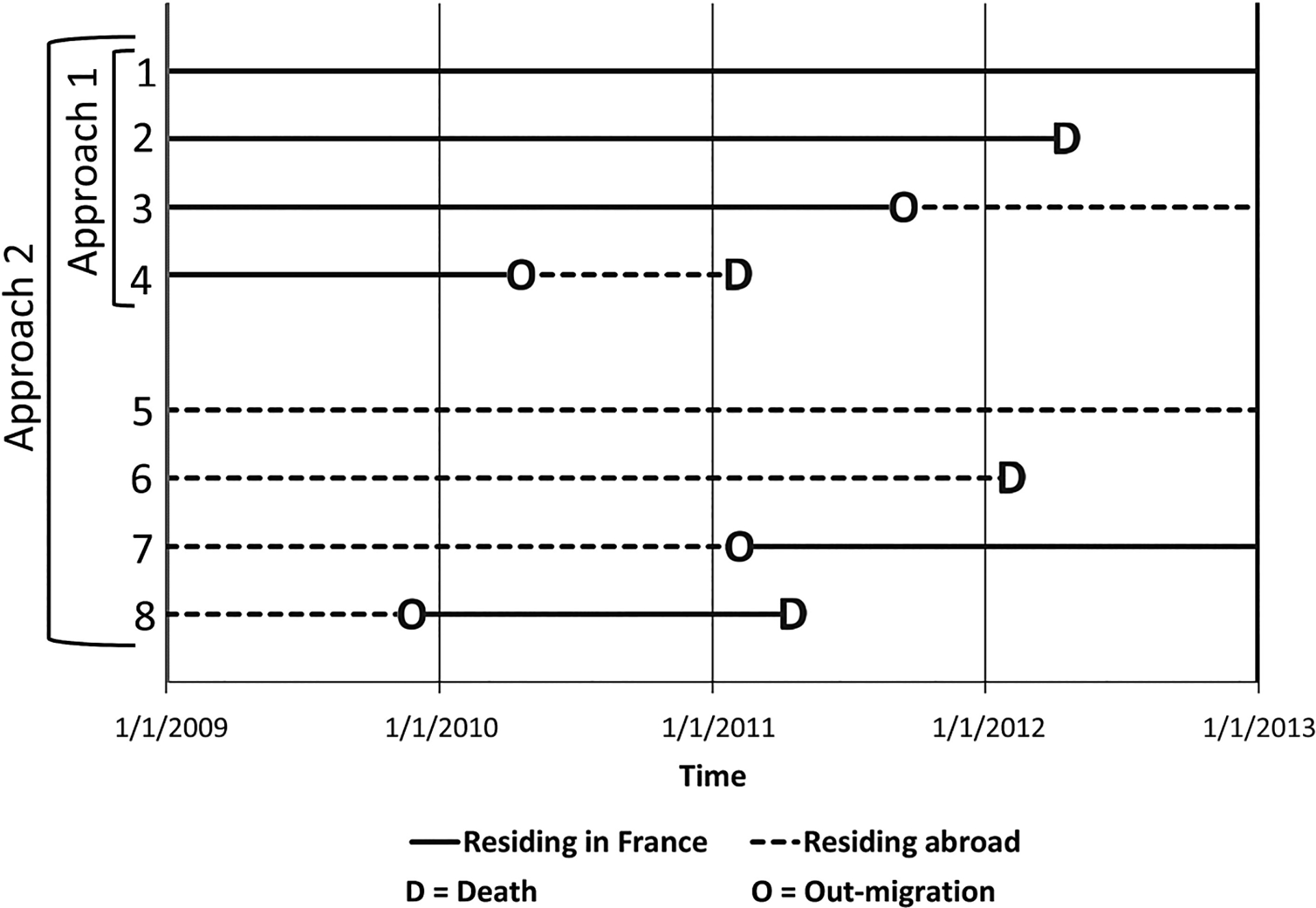
Configuration of life lines of pensioners in the CNAV dataset according to their residence and mortality history. Brackets for Approach 1 and Approach 2 show the types of pensioners included in each analytic approach used in the article.

After performing these simplifications, we obtained eight possible configurations of individual migration and mortality histories. These combinations are represented in Figure 1, with each line representing the lifeline of a pensioner of a given type between January 1, 2009, and January 1, 2013. Lines 1–4 represent pensioners residing in France at baseline. Among them, Lines 1 and 2 represent pensioners who remained in France during the follow-up period: Pensioner 1 was still alive on January 1, 2013, and Pensioner 2 died (in France) before January 1, 2013. Lines 3 and 4 represent pensioners who left France during the follow-up period: Pensioner 3 was still alive on January 1, 2013, and Pensioner 4 died (abroad) before January 1, 2013. Lines 5–8 represent the life lines of pensioners residing abroad at baseline, organized by whether they remained abroad (Lines 5 and 6) or returned to France (Lines 7 and 8) and by whether they remained alive (Lines 5 and 7) or died (Lines 6 and 8). Everyone in our sample falls into one of these eight categories.

In our data, out-migrations from France among the foreign-born almost always (i.e., in 94.7% of cases) correspond to out-migrations to the country of birth rather than to a third country. (See online appendix Table A3 for further details.) In the remainder of the article, we thus do not distinguish between foreign countries of destination and interpret out-migrations from France among the foreign-born as return migrations.

### Methods

Our assessment of return migration selection and its impact on the migrant mortality advantage is primarily based on models that compare mortality for return migrants versus migrants who remained in France. Similar to Turra and Elo’s (2008) study, this approach relies on the prediction that if negative health selection at return is occurring, return migrants will have elevated mortality compared with stayers. However, elevated post-return mortality may arise from factors other than health selection, such as the causal effect of return migration itself and arrival in a place with potentially worse health conditions. To better ascertain the effect of health selection while accounting for the relatively young ages at which many returns occur (as we demonstrate later), we use two analytic approaches.

#### Approach 1

In the first approach, we focus on foreign-born pensioners residing in France at baseline (Lines 1–4 in Figure 1). We follow them prospectively until the end of the follow-up period to determine whether out-migrating from France is associated with higher subsequent mortality relative to remaining in France, as predicted by negative return migration selection. We use a classic, semiparametric Cox proportional hazard model with mortality as the outcome and out-migration as a time-varying explanatory variable:

(1) $$\mu_{x}(y)=\mu_{0}(x+y)\text{exp}[bZ(y)],$$

where μ_*x*_(*y*) is the risk of death at age *x* + *y* for those age *x* at baseline (January 1, 2009), μ_0_(*x* + *y*) is an unspecified baseline hazard, and *Z*(*y*) is a time-varying variable that switches from 0 to 1 when the pensioner moves abroad from France during the follow-up period. We apply this model to foreign-born pensioners, stratified by country of birth.

We then examine whether patterns of post-return mortality documented via Eq. (1) explain the migrant mortality advantage. We use two models to compare the mortality of foreign-born versus native-born pensioners during 2009–2012, also focusing on individuals residing in France at baseline (Lines 1–4 in Figure 1). In Model 1, we focus on the mortality of foreign-born versus native-born pensioners *residing in France*, using a Cox proportional hazard model in which foreign-born pensioners who out-migrated are censored (as they should be) at the time of their out-migration:

(2) $$\mu_{x}(y)=\mu_{0}(x+y)\text{exp}[bX],$$

with **X** representing a vector of dummy variables to identify groups according to their country of birth. This first model allows us to evaluate the extent of the MMA among pensioners in France. Model 2 is similar to Model 1, with one important difference: foreign-born pensioners who out-migrated during the follow-up period are not censored. Instead, they are retained in the exposure pool through the end of the follow-up period, and their deaths occurring abroad are included in the estimation of mortality hazard ratios. This second model allows us to evaluate what the level of the MMA would have been had returning pensioners stayed in France while retaining their observed age at death. The comparison of the relative mortality of foreign-born pensioners in the second versus the first model provides our basis for evaluating the extent to which the MMA may be explained by return migration selection.

#### Approach 2

In the second approach, our analyses use information about the mortality of *all* foreign-born pensioners, including those already residing abroad at baseline. We first examine whether foreign-born pensioners residing abroad experienced higher mortality than those residing in France. We use a Cox proportional model that follows the same equation as in Approach 1 (Eq. (1)), this time applied to the entire sample (Lines 1–8). We then examine the MMA in France and the extent to which it is explained by return migration selection using Models 3 and 4, which are identical to Models 1 and 2 except that they are applied to the entire sample (Lines 1–8).

The advantage of the first approach is that it uses information on the timing of out-migration and relies on a relatively short time window—no more than four years—between out-migration and mortality. As a result, the potential confounding impact of post-remigration conditions in the country of origin can play only a limited role, taking us as close as possible to ascertaining actual selection effects. The second approach, by contrast, will be less directly interpretable in terms of selection effects because of the longer time window between return migration (which, for Lines 5–8, occurred before the start of the follow-up period) and mortality. Its strength, however, is that it considers the large share of foreign-born pensioners who left France at younger ages and may also have been negatively selected. Both approaches have complementary strengths and limitations; in combination, they provide a more comprehensive picture of return migration selection and its impact on the MMA.

## Results

Table 1 shows information about the subsample of individuals who resided in France at baseline (January 1, 2009): those illustrated with Lines 1–4 in Figure 1. This subsample is used in Approach 1. The first column of Table 1 shows the counts of foreign-born pensioners at baseline by region/country of birth, as well as all foreign-born and native-born pensioners. Consistent with France’s immigration history, the regions most represented among these pensioners are North Africa (Algeria, Morocco, and Tunisia) and southern Europe (Italy, Portugal, and Spain). For these two regions, we show details by individual country of birth. The remaining columns show counts of deaths and out-migrations among these pensioners during the follow-up period (2009–2012). Of the 83,658 foreign-born pensioners residing in France at baseline, 12,592 (15.1%) died in France, and 2,435 (2.9%) out-migrated during the follow-up period. Among those who out-migrated, 437 (17.9%) died between the time of the relocation abroad and the end of the follow-up period.

These data are used in the prospective model for Approach 1, with hazard ratios representing whether foreign-born pensioners who out-migrated had higher subsequent mortality than those who remained in France. Results presented in Table 2 show that return migration is associated with substantial excess mortality. For all countries of birth combined, the mortality hazard ratio is 2.406 (*p* < .001). This excess mortality is highly consistent among migrant groups. The only foreign-born pensioners with a statistically nonsignificant hazard ratio are those born in the residual “other foreign countries” category, a small group of pensioners with very few (11) returnees during the follow-up period. All other groups display statistically significant hazard ratios ranging from 1.556 to 3.617, with no clear pattern by region/country of birth. Pensioners born in and returning to low-mortality regions/countries (e.g., Italy, Portugal, and Spain) do not have lower hazard ratios associated with return migration.

Is this excess mortality among returnees strong enough to explain the migrant mortality advantage? We answer this question by comparing mortality among foreign-born versus native-born pensioners in Models 1 and 2; results are shown in Table 3. Model 1, which censors pensioners who out-migrate at the time of out-migration, confirms a consistent migrant mortality advantage at ages 65+ in France. With a hazard ratio of 0.927 (*p* < .001), foreign-born pensioners residing in France have mortality risks at ages 65+ that are 7.3% lower, on average, than those of their native-born counterparts. This mortality advantage is particularly pronounced among pensioners from Morocco (hazard ratio = 0.845, *p* < .001), Tunisia (0.880, *p* < .001), and Portugal (0.883, *p* < .001). The MMA was not shared by all groups of foreign-born pensioners, however. Pensioners born in Italy, other countries in Europe, and other countries in Africa did not have statistically significant hazard ratios.

Model 2, which does not censor foreign-born pensioners who out-migrated during the follow-up period but instead keeps them in the exposure pool and takes their deaths abroad into account, seeks to measure the effect of return migration selection on the MMA. Results show that the hazard ratios are higher in Model 2 than in Model 1. This finding is expected because Model 2 includes the mortality of returnees, who had higher mortality than those who stayed. The effects are relatively minor, however. When we examine all foreign-born pensioners combined, the hazard ratio increases only slightly, from 0.927 to 0.946, and retains its significance. Effects vary by country of birth, with two countries (Algeria and Portugal) experiencing larger increases in the hazard ratio than others. Overall, though, the effects are modest. Among countries with a statistically significant MMA, only one (Spain) loses significance once mortality among returnees is considered. Regions/countries with no statistically significant hazard ratio in Model 1 remain the same in Model 2. The overall lesson of this comparison is that even though we detect strong excess mortality among foreign-born pensioners who resided abroad, this excess mortality appears to explain only a small portion of the overall MMA. This result is due to the relative rarity of return migration among these pensioners. In our sample, only 2.9% of those residing in France at baseline out-migrated during the follow-up period. Even though these returnees experienced substantial excess mortality, their demographic weight is too small to reverse the advantage experienced by the overwhelming majority of those who stayed.

**Fig. 2:**
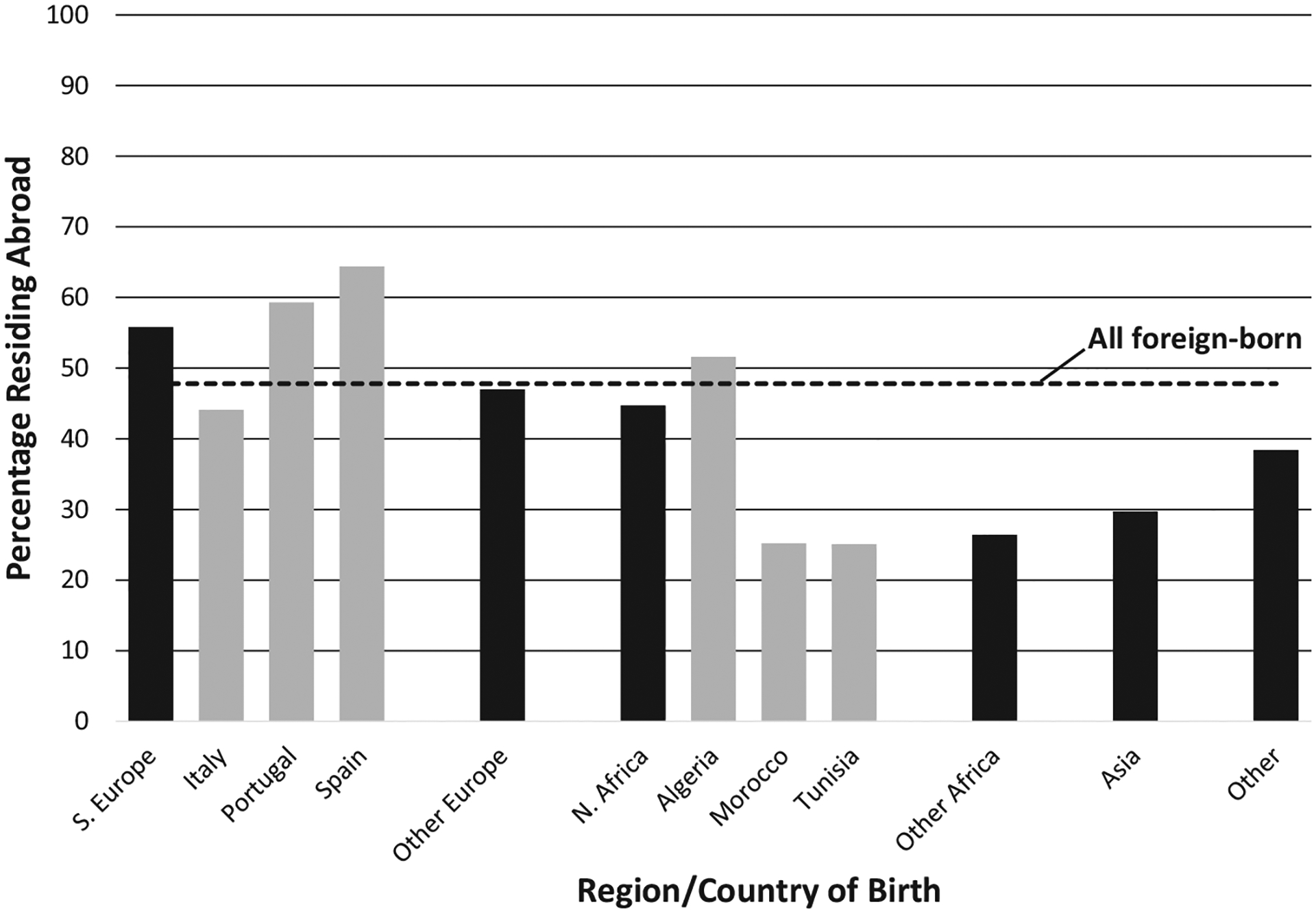
Percentage of CNAV foreign-born male pensioners aged 65+ residing abroad as of January 1, 2009, by region/country of birth

Approach 1 excluded pensioners who had returned before 2009 (Lines 5–8 in Figure 1). Table 4 and Figure 2 show that this group of early returnees represents a large share of foreign-born pensioners, with almost half (47.8%) residing abroad in 2009. This reality illustrates the importance of return migration across the life course in a receiving country like France: by the time they reach retirement age, almost half of foreign-born males who had worked in France at some point had already left France and were receiving their French pension abroad. The CNAV data offer a rare opportunity to capture this combined stock of current and former immigrants. Table 4 and Figure 2 also show variations in the proportion of returnees by region/country of birth. The proportions tend to be higher among pensioners from neighboring EU countries, where return migration is easier. For example, 64.4% of Spain-born pensioners and 59.3% of Portugal-born pensioners resided abroad at baseline. At the other end of the spectrum, countries such as Morocco and Tunisia, for which return migration may be more costly, have smaller proportions of returnees (25.2% and 25.1%, respectively). An analysis of sequences of pension contributions in the CNAV data suggests that a large share of returns occur in midlife, at around ages 45–55, with a smaller peak around retirement (Gansey et al. 2019).

Approach 2 accounts for this large stock of individuals already residing abroad at baseline. Table 5 compares the mortality risks of foreign-born pensioners residing abroad versus those residing in France. Results are consistent with the patterns found in Table 2 (for Approach 1): mortality rates are higher among foreign-born pensioners residing abroad than among those residing in France. Hazard ratios are systematically greater than 1, with statistical significance reached for all regions except the small “other foreign countries” residual category, all but one of the top six countries of origin (Tunisia), and all foreign-born pensioners combined (hazard ratio = 1.159, *p* < .001). Including early returnees does not modify our earlier conclusion that residing abroad versus in France is associated with higher mortality at ages 65+. The hazard ratios in Table 5 are not as high as those generated with Approach 1. The consistency of the pattern of excess mortality across foreign-born pensioners with countries of origin as diverse as Italy, Morocco, Portugal, and Spain is nonetheless striking.^1^

As with Approach 1, we examine the extent to which the excess mortality associated with residing abroad may explain the MMA. Model 3 in Table 6 compares the mortality of foreign-born versus native-born pensioners residing in France. This model is similar to Model 1 in Table 3 but considers all deaths and exposures associated with a French residence, regardless of whether pensioners resided in France or abroad at baseline. Results are very similar to those from Model 1, which is expected because the main difference between the models arises from a small number of pensioners who resided abroad at baseline but returned to France during the follow-up period. The hazard ratio of 0.926 for all foreign-born pensioners combined in Model 3 reflects the same overall mortality advantage as in Model 1 (0.927). Hazard ratios for individual groups of pensioners by region/country of residence are virtually identical to those estimated in Model 1.

The main difference from Approach 1 appears in Model 4, which examines the relative mortality of foreign-born pensioners (vs. their native-born counterparts) regardless of their country of residence at baseline or during the follow-up period. Including the large stock of foreign-born pensioners already residing abroad at baseline provides a different picture. For all countries of birth combined, the hazard ratio rises from 0.926 to 0.998 and loses significance, indicating that the MMA disappears once we include foreign-born pensioners residing abroad in the comparison. We find this pattern of lost advantage among pensioners from several regions/countries of birth, including Algeria and Portugal. In addition, certain groups of foreign-born pensioners that did not have an MMA in Model 3 (i.e., those born in Italy and other European countries) exhibit statistically significant excess mortality once the mortality of all foreign-born pensioners is considered in Model 4. The overall lesson of comparing Model 4 with Model 3 is that the MMA disappears (or is sometimes reversed) once the mortality of foreign-born pensioners who lived abroad at baseline is considered. This finding results from the combined effect of large proportions of foreign-born pensioners living abroad and their higher mortality relative to those residing in France—a systematic pattern across a wide range of countries of origin. Although the excess mortality associated with foreign residence is larger in Approach 1, effects on the MMA are stronger in Approach 2.

## Discussion

During their employment in a receiving country, foreign-born workers accumulate rights to a pension they can receive upon reaching retirement age, regardless of where they reside. Some of these workers return to their country of origin while relatively young, after only a few years of employment in the receiving country. Some wait until or after retirement to return. Some never return, staying in the receiving country until the end of their life. Comparing the mortality of resident foreign-born versus native-born individuals in receiving countries ignores these returns, even though in a country like France, roughly half the initial stock of foreign-born male workers return to their country of origin by retirement age. Those still residing in France at older ages experience a consistent migrant mortality advantage relative to natives. This advantage, however, is difficult to interpret in a context where mortality rates are calculated based on only a portion of the initial stock of foreign-born workers, ignoring the experience of those who returned. If returns were selective, those remaining in France might represent the healthiest of the initial stock of immigrants.

In this study, we take advantage of a unique, longitudinal source of information that allows us to observe the mortality of males aged 65+ who were immigrant workers in France and returned to their country of origin before or after retirement. We find that foreign-born pensioners residing abroad experience higher mortality than their peers staying in France. This excess mortality is particularly strong among recent returnees.

We interpret the excess mortality of recent returnees, shown in Approach 1, as indicating a strong and pervasive process of negative return migration selection. For these individuals, return migration occurred after age 65, and this excess mortality was observed over a short time window following return migration. This short time window attenuates the possible confounding effect of exposures to conditions in the country of origin after the return. Moreover, the excess mortality associated with returning to the country of origin is observed across diverse countries of origin, including countries with mortality conditions and health care systems comparable to those in France (e.g., Italy and Spain). This finding further supports our interpretation that excess mortality results from negative selection rather than differences in mortality conditions or health care systems between France and the country of origin.

Conditions in the country of origin after return are likely more influential for the mortality of those who returned before retirement. Indeed, some of the pensioners examined in Approach 2 may have returned years before retiring, and thus their excess mortality observed at older ages is, *a priori*, less directly interpretable in terms of negative return selection. Nonetheless, like in the case of Approach 1, our results are strikingly consistent across diverse countries of origin. We find excess mortality even among immigrants returning to Italy, Portugal, and Spain—countries with mortality conditions that are not particularly different from those in France. In 2010–2014, male life expectancy at age 65 in Italy and Spain was 18.62 years and 18.64 years, respectively, versus 18.92 years in France (Human Mortality Database 2021). These life expectancies translate to mortality ratios of 1.016 for Italy and 1.015 for Spain (vs. France), which are substantially lower than those documented in Table 5 for these two countries of birth. The mortality gap is slightly larger in Portugal, with a life expectancy at age 65 of 17.60 years. Here also, this difference in life expectancy translates to a much lower mortality ratio than that found in Table 5: 1.075 versus 1.227 in Table 5. The consistency of the pattern of excess mortality shown in Table 5, even for countries of origin that do not exhibit important mortality differences from France, supports the conclusion that the excess mortality arises from negative return selection.

The higher mortality hazard ratios produced from Approach 1 relative to Approach 2 further support this interpretation of our results. In Approach 1, post-return exposure to conditions in the country of origin is shorter than in Approach 2, yet returnees experience higher relative mortality. If exposure to worse mortality conditions in the country of origin were the dominant explanation, we would observe lower hazard ratios in Approach 1 than in Approach 2. It is possible that negative return migration selection is just as high in Approach 1 as in Approach 2 but that the impact of negative selection in Approach 2 is attenuated by some positive consequences of return migration on health. Our data do not allow us to make this distinction, but if this were the case, then Approach 2 would underestimate the true scale of negative selection return, and our results would be conservative.

Our results extend the literature in several ways. First, we expand the findings of Turra and Elo (2008) via a similar approach applied to the European context. Like Turra and Elo, we find that return migrants experience higher post-remigration mortality than comparable migrants who stayed, with particularly high excess mortality among recent returnees (Approach 1). A key difference is that once we account for mortality among all return migrants regardless of age at return (Approach 2), the MMA essentially disappears in our study; by contrast, in the Turra and Elo study, the MMA remained unaffected. This difference arises primarily from the much more frequent return in the French context. In the U.S. Social Security data Turra and Elo used, 9.3% of foreign-born Hispanic males and 11.0% of non-Hispanic White males resided abroad as pensioners, compared with 47.8% of foreign-born males in the French CNAV data. Taking the excess mortality of return migrants into account is therefore much more consequential in the French context.

Overall, our results from Approach 2 emphasize the importance of negative return migration selection as a mechanism that may operate not only at older ages, as suggested in much of the salmon bias literature, but across the life course. Our results do support the operation of negative return selection at older ages, but they also suggest that negative return selection is likely to occur over the life course, with mortality impacts that remain visible years later. Although return migration selection across the life course was previously theorized and documented, our results show that the dominant selection process appears to be negative rather than positive. This finding is consistent with those of several studies focusing on health outcomes among working-age return migrants, with results indicating the presence of negative health selection (Arenas et al. 2015; Lu and Qin 2014; Martinez-Cardoso and Geronimus 2021). It is also consistent with the hypothesis initially proposed by Razum et al. (1998), who emphasized negative selection via SES; they postulated that immigrants who fail to cope well socially and economically in the receiving country might remigrate “even before becoming manifestly ill” (Razum et al. 1998:302).

Regardless of whether return migration is motivated by health or other factors, our study stresses the importance of considering returns occurring at relatively young ages when examining return migration selection and its impact on the MMA or the immigrant health advantage (Arenas et al. 2015; Riosmena et al. 2017). The impact of return migration selection effects on the MMA may be larger than previously suggested, especially in contexts with a large volume of return migration, such as France.

Our results show that the migrant mortality advantage at ages 65+ is reduced or eliminated once return migration selection is considered. From a life course perspective, the migrant mortality advantage in receiving countries tends to be largest around age 45 and then gradually diminishes with age via a process of mortality convergence (Guillot et al. 2018; Namer and Razum 2018). By the time immigrants reach retirement age, little advantage remains, as illustrated by the foreign-born versus native-born mortality hazard ratio of 0.926 shown in Table 6. By contrast, mortality ratios observed around age 45 in several receiving countries, including France, are 0.6–0.7 (Guillot et al. 2018). Our results for mortality at ages 65+ suggest that return migration selection *slows* the process of mortality convergence with age. Were returns not selective, the process of mortality convergence with age would likely occur faster than observed using reported mortality data.

Our study has limitations. First, we defined immigrants using country of birth information. This definition is problematic for pensioners born in Algeria because it does not distinguish between former European colonists in Algeria who returned to France when Algeria became independent in 1962 versus Algerian immigrants. In 2019, only 61.3% of the Algeria-born population residing in France had a foreign nationality at birth and were thus considered immigrants. This issue may explain why the migrant mortality advantage is not as large for Algeria as for the other two North African countries (Morocco and Tunisia). Using country of birth information as the sole criteria for defining immigrants is likely to have a negligible impact on the results for the other countries of birth, for which the proportion of true immigrants among the foreign-born population is 82% to 86% (INSEE 2022a).

Second, our data do not include information on returnees who never claimed their right to a pension. We do not have information about how frequently this occurs, but it is likely to be more common among returnees who spent short amounts of time in France, for whom pension amounts may be trivial. To address potential selection biases associated with pension-claiming, we conducted a sensitivity analysis in which we removed from the sample those returnees with fewer than five years of salary contributions in France. The results are virtually unchanged (see Tables A6 and A7, online appendix), suggesting that such selection biases are likely to be small.

Third, our data do not include information on pensioners who never worked in the private sector. This omission is likely to have only a negligible impact on our results because of the very high coverage among males in the CNAV database, as discussed in the Data section. Our data also omit information on workers who spent their entire time in France without a regular work contract. However, undocumented immigrants in France can and often do work with official contracts because proof of employment and tax returns can be used as positive evidence for legalization. This path to legalization for undocumented immigrants in France also implies that very few immigrants who still reside in France at retirement age can be expected to have remained undocumented. Workers who spent their entire time in France without a work contract are likely to have resided in France for only short durations. As shown in our sensitivity analyses discussed earlier (Tables A6 and A7), excluding individuals with short durations of stay has virtually no effect on the results.

Fourth, deaths abroad may be underreported or reported late. Our study addressed this issue by considering information on pension payments, which are conditional on producing a certificate of life. However, mortality abroad remains likely to be underreported, especially at very old ages, at which observed slopes of age-specific mortality rates appear implausibly low even after our mortality adjustment (results not shown). This pattern implies that our estimates of excess mortality associated with residing abroad remain underestimated. Our results are thus likely conservative and underestimate the true level of negative return migration selection.

Fifth, the residence information we use to distinguish between pensioners residing in France and those living abroad is based on declared rather than actual residence. Some pensioners who declare French residence might spend most (if not all) of their time abroad, given that retaining an official residence in France brings some advantages in terms of pension amounts and health care coverage. This issue would affect our conclusion only if the pensioners who declared residing in France but actually resided abroad had lower mortality risks than those who actually resided in France. There is no reason to believe that such positive selection occurs. On the contrary, it is likely that negative return selection also operates for unofficial returns, in which case our results would be conservative, underestimating the amount of negative return selection.

Finally, our analytic approach does not allow us to rule out the possibility that at least some excess mortality occurring after returning to the country of origin may be *caused* by return migration rather than solely arising from negative selection. For a country of origin with worse health conditions and poorer health care, return migration may lead to excess mortality even without selection. Because our data do not include information on health or SES before remigration, we cannot evaluate the respective roles of selection and causal effects in explaining the excess mortality of return migrants. As discussed earlier, we nonetheless conclude that our results are best explained by selection because excess mortality is present across a wide range of countries of origin, including countries with mortality conditions similar to those of France. Moreover, excess mortality is particularly strong among recent migrants (Approach 1), for whom post–return migration conditions are less likely to play a substantial role. For a more direct ascertainment of selection versus causation in explaining the excess mortality of returnees, future research would benefit from using longitudinal data that would not only track the mortality of immigrants after they return to their country of birth but also provide sufficient information on their characteristics during the period preceding their return.

## Supplementary Material

Online Appendix

## Figures and Tables

**Table 1:** Distribution of CNAV foreign-born male pensioners aged 65+ residing in France on January 1, 2009, and their worldwide deaths and out-migrations until December 31, 2012, by country of birth

Country of Birth	Residing in France on January 1, 2009 (Lines 1–4)^a^	Died While Residing in France, 2009–2012 (Line 2)^a^	Out-migrated From France, 2009–2012 (Lines 3–4)^a^	Died After Out-migrating From France, 2009–2012 (Line 4)^a^
Southern Europe	24,371	4,260	493	77
Italy	10,493	2,155	65	20
Portugal	7,644	877	283	40
Spain	6,234	1,228	145	17
Other Countries in Europe (including Russia)	7,095	1,532	91	12
North Africa	45,770	5.994	1,738	318
Algeria	29,602	4,073	1,179	247
Morocco	9,459	1,056	363	46
Tunisia	6,709	865	196	25
Other Countries in Africa	2,556	294	38	16
Asia (including Turkey)	3,050	395	64	12
Other Foreign Countries (America, Oceania)	816	117	11	2
All Foreign-born	83,658	12,592	2,435	437
Native-born	32,275	5,826	15	3

a Typologies of residence and mortality follow-up are depicted in Figure 1.

**Table 2:** Effect of out-migration (ref. = remaining in France) on subsequent mortality among CNAV foreign-born male pensioners aged 65+ residing in France on January 1, 2009, and followed up until December 31, 2012, by country of birth: Cox regression models with associated 95% confidence intervals (CIs)

Country of Birth	Mortality Hazard Ratio	95% CI
Southern Europe	2.046***	1.632–2.564
Italy	3.040***	1.954–4.730
Portugal	2.014***	1.460–2.777
Spain	1.659*	1.026–2.683
Other Countries in Europe (including Russia)	1.556***	1.632–2.564
North Africa	2.528***	2.258–2.831
Algeria	2.568***	2.257–2.922
Morocco	2.429***	1.806–3.268
Tunisia	2.295***	1.540–3.422
Other Countries in Africa	2.528***	2.258–2.831
Asia (including Turkey)	3.617***	1.994–6.560
Other Foreign Countries (America, Oceania)	3.529	0.846–14.718
All Foreign Countries	2.406***	2.187–2.647

*Note*: Models are stratified by country of birth, with country of residence (abroad vs. France) treated as a time-varying variable.

* *p* < .05;

*** *p* < .001

**Table 3:** Mortality hazard ratios of CNAV foreign-born versus native-born male pensioners aged 65+ residing in France on January 1, 2009, and followed up until December 31, 2012, by country of birth: Cox regression models with associated 95% confidence intervals (CIs)

	Model 1: Foreign-born Pensioners Residing in France		Model 2: All Foreign-born Pensioners, Including Those Who Out-migrated in 2009–2012	
Country of Birth	Hazard Ratio	95% CI	Hazard Ratio	95% CI
France (ref.)^a^	—	—	—	—
Southern Europe	0.958*	0.921–0.996	0.967	0.929–1.005
Italy	1.004	0.955–1.055	1.010	0.961–1.061
Portugal	0.883***	0.823–0.949	0.905**	0.843–0.970
Spain	0.939*	0.883–0.999	0.944	0.887–1.003
Other Countries in Europe (including Russia)	1.021	0.965–1.081	1.024	0.968–1.083
North Africa	0.891***	0.859–0.924	0.919***	0.887–0.953
Algeria	0.905***	0.869–0.942	0.938**	0.902–0.976
Morocco	0.845***	0.791–0.903	0.867***	0.812–0.925
Tunisia	0.880***	0.819–0.945	0.895**	0.834–0.960
Other Countries in Africa	0.905	0.804–1.017	0.947	0.844–1.062
Asia (including Turkey)	0.885*	0.799–0.980	0.904*	0.817–1.000
Other Foreign Countries (America, Oceania)	0.808*	0.673–0.971	0.819**	0.683–0.982
All Foreign Countries	0.927***	0.899–0.956	0.946***	0.917–0.975

*Notes*: Model 1: Foreign-born pensioners who out-migrated from France in 2009–2012 are censored at out-migration. Their exposures and deaths after out-migration are not included in the estimation of mortality hazard ratios. Model 2: Foreign-born pensioners who out-migrated from France in 2009–2012 remain in the exposure pool through the end of the observation period. Their worldwide deaths are included in the estimation of mortality hazard ratios.

a The reference category in both models is native-born pensioners residing in France. The 15 native-born pensioners who out-migrated in 2009–2012 are censored at out-migration.

* *p* < .05;

** *p* < .01;

*** *p* < .001

**Table 4:** Distribution of CNAV male pensioners aged 65+ by country of birth and place of residence on January 1, 2009, and their subsequent worldwide deaths until December 31, 2012, by country of birth and place of residence at the time of death

	Pensioners on January 1, 2009				Deaths 2009–2012		
	Place of Residence on January 1, 2009				Place of Residence at Death		
Country of Birth	France	Abroad	Total	% Residing Abroad	France	Abroad	Total
Southern Europe	24,371	30,716	55,087	55.8	4,271	5,402	9,673
Italy	10,493	8,262	18,755	44.1	2,157	1,614	3,771
Portugal	7,644	11,153	18,797	59.3	884	1,985	2,869
Spain	6,234	11,301	17,535	64.4	1,230	1,803	3,033
Other Countries in Europe (including Russia)	7,095	6,291	13,386	47.0	1,535	1,297	2,832
North Africa	45,770	37,035	82,805	44.7	6,018	6,644	12,662
Algeria	29,602	31,595	61,197	51.6	4,091	5,621	9,712
Morocco	9,459	3,191	12,650	25.2	1,060	654	1,714
Tunisia	6,709	2,249	8,958	25.1	867	369	1,236
Other Countries in Africa	2,556	916	3,472	26.4	294	144	438
Asia (including Turkey)	3,050	1,287	4,337	29.7	396	240	636
Other Foreign Countries (America, Oceania)	816	509	1,325	38.4	117	86	203
All Foreign-born	83,658	76,754	160,412	47.8	12,631	13,813	26,444
Native-born	32,275	320	32,595	1.0	5,828	63	5,891
Total	115,933	77,074	193,007	—	18,459	13,876	32,335

**Table 5:** Mortality hazard ratios of CNAV foreign-born male pensioners aged 65+ residing abroad (i.e., out-migrated) versus in France (the reference category), 2009–2012, by country of birth: Cox regression models with associated 95% confidence intervals (CIs)

Country of Birth	Mortality Hazard Ratio	95% CI
Southern Europe	1.117***	1.072–1.163
Italy	1.098**	1.029–1.173
Portugal	1.227***	1.132–1.330
Spain	1.087*	1.008–1.172
Other Countries in Europe (including Russia)	1.251***	1.158–1.350
North Africa	1.176***	1.135–1.218
Algeria	1.141***	1.096–1.188
Morocco	1.476***	1.336–1.631
Tunisia	1.105	0.978–1.249
Other Countries in Africa	1.287*	1.052–1.574
Asia (including Turkey)	1.806***	1.528–2.135
Other Foreign Countries (America, Oceania)	1.215	0.914–1.614
All Foreign Countries	1.159***	1.131–1.187

*Note*: Models are stratified by country of birth, with country of residence (abroad vs. France) treated as a time-varying variable.

* *p* < .05;

** *p* < .01;

*** *p* < .001

**Table 6:** Mortality hazard ratios of CNAV foreign-born versus native-born male pensioners aged 65+, 2009–2012, by country of birth: Cox regression models with associated 95% confidence intervals (CIs)

	Model 3: Foreign-born Pensioners Residing in France		Model 4: All Foreign-born Pensioners, Including Those Residing Abroad	
Country of Birth	Hazard Ratio	95% CI	Hazard Ratio	95% CI
France (ref.)^a^	—	—	—	—
Southern Europe	0.960*	0.923–0.999	1.013	0.981–1.047
Italy	1.007	0.958–1.058	1.043*	1.001–1.087
Portugal	0.881***	0.821–0.946	1.019	0.974–1.066
Spain	0.944	0.888–1.004	0.975	0.933–1.019
Other Countries in Europe (including Russia)	1.030	0.974–1.090	1.138***	1.088–1.190
North Africa	0.887***	0.856–0.920	0.960**	0.930–0.990
Algeria	0.903***	0.867–0.940	0.972	0.941–1.004
Morocco	0.839***	0.786–0.896	0.939*	0.890–0.991
Tunisia	0.877***	0.817–0.942	0.904**	0.850–0.961
Other Countries in Africa	0.899	0.800–1.011	0.962	0.873–1.061
Asia (including Turkey)	0.883*	0.798–0.978	1.066	0.982–1.157
Other Foreign Countries (America, Oceania)	0.811*	0.675–0.974	0.870	0.757–1.001
All Foreign Countries	0.926***	0.898–0.956	0.998	0.970–1.027

*Notes*: Model 3 accounts for exposures and deaths occurring while residing in France only. Model 4 accounts for exposures and deaths among all CNAV pensioners, regardless of place of residence (in France or abroad).

a The reference category in both models involves native-born pensioners residing in France.

* *p* < .05;

** *p* < .01;

*** *p* < .001

## Footnotes

1 Results in Tables 2 and 5 remain virtually identical when we focus only on foreign-born out-migrants who returned to their country of birth (i.e., excluding those who out-migrated to other foreign countries), confirming that our results also apply specifically to the process of return migration. See Tables A4 and A5 (online appendix) for details.
