# Supplementary material for: Return Migration Selection and Its Impact on the Migrant Mortality Advantage: New Evidence Using French Pension Data
Source: Demography. Author manuscript; Available in PMC 2023 Oct 20. (PMC10587819; doi:10.1215/00703370-10938784)
Supplement: Online Appendix [file NIHMS1936130-supplement-Online_Appendix.pdf]

Online Appendix

Return Migration Selection and Its Impact on the Migrant Mortality Advantage:

New Evidence Using French Pension Data

Michel Guillot, Myriam Khlat, Romeo Gansey, Matthieu Solignac, and Irma Elo

Table A1: Typologies of missing residence information and actions taken for imputation

Typology	Number of Quarters with Missing Information				Total	Percent	Example	Action
	1	2	3	4+				
Sequence of F's & Sequence of M's & death in France	401	417	464	45	1,327	52.6	FFFFFFFFMM	M--->F
Sequence of E's & Sequence of M's & death abroad or missing	328	291	322	2	943	37.4	EEEEEEEMM	M--->E
Sequence of F's & Sequence of M's & death abroad or missing	61	58	83	2	204	8.1	FFFFFFFFMM	M--->F
Sequence of E's and F's and M's & death abroad or missing	4	6	4	0	14	0.6	EEEFFFFMM	M--->F
Sequence of M's & Death in France	3	0	0	5	8	0.3	MMM	M--->F
Concordant Sequence of E's & M's & E's	3	2	1	1	7	0.3	EEMMMEEE	M--->E
Sequence of M's & Sequence of E's	3	0	2	2	7	0.3	MEEEEEEE	M--->E
Concordant Sequence of F's & M's & F's	2	0	1	1	4	0.2	FFFMMFFFF	M--->F
Sequence of E's & Sequence of M's & death in France	2	0	1	0	3	0.1	EEEEEEEMM	M--->F
Sequence of E's & Sequence of M's & alive	0	1	0	1	2	0.1	EEEEEEEMM	M--->E
Sequence of F's and E's and M's & death in France	0	1	0	0	1	0.0	FFFEEEEMM	M--->F
Sequence of E's and F's and M's & death in France	0	1	0	0	1	0.0	EEEFFFFMM	M--->F
Sequence of E's and F's and M's & alive	0	0	0	1	1	0.0	EEEFFFFMM	M--->F
Various Sequences of F's/E's & ending with Sequence of M's & death abroad or missing	0	0	1	0	1	0.0	EEFFEEEMM	M--->E
**Total**	**807**	**777**	**879**	**60**	**2,523**	**100.0**		

Notes:
M = Missing
F = Residence in France
E = Residence abroad
---> means “is replaced with”

Table A2: Rule for imputing deaths abroad based on pension sequences observed among male pensioners who resided abroad when they last received a pension

Year						Count of Pensioners	Action
**2009**	**2010**	**2011**	**2012**	**2013**	**2014**		
1	0	0	0	0	0	159	impute death on 7/1/2009
1/0	1	0	0	0	0	237	impute death on 7/1/2010
1/0	1/0	1	0	0	0	263	impute death on 7/1/2011
1/0	1/0	1/0	1	0	0	300	impute death on 7/1/2012
**Total**						**959**	

Notes:
1 = pension received
0 = no pension received
1/0 = 1 or 0

Table A3: Distribution of CNAV male foreign-born pensioners aged 65+ by region of birth of birth and place of residence 1/1/2009

	Place of residence on 1/1/2009				
Region of birth	France	Abroad	Abroad in country of birth	Abroad elsewhere	% residing in country of birth among those residing abroad
Southern Europe	24,371	30,716	29,414	1,032	95.8%
Other countries of Europe (incl. Russia)	7,095	6,291	5,132	1,159	81.6%
North Africa	45,770	37,035	35,920	1,115	97.0%
Other countries of Africa	2,556	916	729	187	79.6%
Asia (incl. Turkey)	3,050	1,287	1,070	217	83.1%
Other Foreign Countries (America, Oceania)	816	509	453	56	89.0%
**All foreign born**	**83,658**	**76,754**	**72,718**	**4,036**	**94.7%**

Table A4: Effect of out-migration to the country of birth on subsequent mortality among CNAV foreign-born male pensioners aged 65+ residing in France on 1/1/2009 and followed-up until 12/31/2012, by country of birth. Cox regression models with associated 95% CI.

**Country of birth**		**Mortality Hazard Ratio**	**95% CI**
Southern Europe	Remained in France (Ref.)	–	
	Out-migrated	2.096***	[1.673 - 2.627]
Other Countries of Europe (incl. Russia)	Remained in France (Ref.)	–	
	Out-migrated	2.862***	[1.615 - 5.072]
North Africa	Remained in France (Ref.)	–	
	Out-migrated	2.637***	[2.355 - 2.953]
Other countries of Africa	Remained in France (Ref.)	–	
	Out-migrated	13.749***	[8.072 - 23.417]
Asia (incl. Turkey)	Remained in France (Ref.)	–	
	Out-migrated	4.042***	[2.227 – 7.337]
Other foreign countries (America, Oceania)	Remained in France (Ref.)	–	
	Out-migrated	4.954	[0.669 - 36.668]
**All Foreign Countries**	**Remained in France (Ref.)**	**–**	
	**Out-migrated**	**2.561*****	**[2.327 - 2.819]**

Note: Models are stratified by country of birth, with country of residence (France vs. country of birth) treated as a time varying variable.

Table A5: Mortality hazard ratios of CNAV foreign-born born male pensioners aged 65+ residing in their country of birth vs. in France, 2009-2012, by country of birth. Cox regression models with associated 95% CI.

**Country of birth**		**Mortality Hazard Ratio**	**95% CI**
Southern Europe	Remained in France (Ref.)	–	
	Out-migrated	1.116***	[1.071 – 1.162]
Other Countries of Europe	Remained in France (Ref.)	–	
	Out-migrated	1.290***	[1.189 – 1.398]
North Africa	Remained in France (Ref.)	–	
	Out-migrated	1.175***	[1.134 – 1.217]
Other countries of Africa	Remained in France (Ref.)	–	
	Out-migrated	1.365**	[1.087 - 1.715]
Asia	Remained in France (Ref.)	–	
	Out-migrated	1.911***	[1.603 - 2.277]
Other foreign countries (America, Oceania)	Remained in France (Ref.)	–	
	Out-migrated	1.203	[0.769 - 1.882]
**All Foreign Countries**	**Remained in France (Ref.)**	**–**	
	**Out-migrated**	**1.158*** **	**[1.130 - 1.187]**

Note: Models are stratified by country of birth, with country of residence (France vs. country of birth) treated as a time varying variable.

Table A6: Mortality hazard ratios of CNAV foreign-born born male pensioners aged 65+ residing abroad vs. in France, 2009-2012, by country of birth. Cox regression models with associated 95% CI. Returnees with less than 5 years of salary contributions in France are excluded from the sample.

**Country of birth**		**Mortality Hazard Ratio**	**95% CI**
Southern Europe	Remained in France (Ref.)	–	
	Out-migrated	1.123***	[1.074 – 1.175]
Other Countries of Europe	Remained in France (Ref.)	–	
	Out-migrated	1.259***	[1.140 – 1.391]
North Africa	Remained in France (Ref.)	–	
	Out-migrated	1.204***	[1.159 – 1.250]
Other countries of Africa	Remained in France (Ref.)	–	
	Out-migrated	1.620**	[1.282 - 2.046]
Asia	Remained in France (Ref.)	–	
	Out-migrated	1.823***	[1.477 - 2.251]
Other foreign countries (America, Oceania)	Remained in France (Ref.)	–	
	Out-migrated	1.106	[0.669 - 1.829]
**All Foreign Countries**	**Remained in France (Ref.)**	**–**	
	**Out-migrated**	**1.171*** **	**[1.140 - 1.203]**

Note: Models are stratified by country of birth, with country of residence (France vs. country of birth) treated as a time varying variable.

Table A7: Mortality hazard ratios of CNAV foreign-born vs. native-born male pensioners aged 65+, 2009-2012, by country of birth. Cox regression models with associated 95% CI. Returnees with less than 5 years of salary contributions in France are excluded from the sample.

Country of birth	Model 3: Foreign-born pensioners residing in France		Model 4: All foreign-born pensioners, including those residing abroad	
	HR	95% CI	HR	95% CI
France (Ref.)*	–	–	–	–
Southern Europe	0.960*	[0.923 – 0.999]	1.006	[0.972 – 1.040]
Other countries of Europe	1.029	[0.973 – 1.089]	1.092***	[1.039 – 1.148]
North Africa	0.890***	[0.858 – 0.923]	0.961*	[0.931 – 0.993]
Other countries of Africa	0.903	[0.803 – 1.016]	0.997	[0.899 – 1.105]
Asia	0.886*	[0.800 – 0.981]	0.995	[0.909 – 1.088]
Other Foreign Countries (America, Oceania)	0.812*	[0.676 – 0.975]	0.822*	[0.693 – 0.975]
All Foreign Countries	0.928***	[0.899 - 0.957]	0.989	[0.961 - 1.018]

Model 3: Accounting for exposures and deaths occurring while residing in France only
Model 4: Accounting for exposures and deaths among all CNAV pensioners, regardless of place of residence (in France or abroad)
* The reference category in both models involves native-born pensioners residing in France.
